# How the cascading effects of a single behavioral trait can generate personality

**DOI:** 10.1002/ece3.1157

**Published:** 2014-07-10

**Authors:** Frédérique Dubois, Luc-Alain Giraldeau

**Affiliations:** 1 Département de Sciences Biologiques, Université de MontréalMontréal, Quebec, Canada; 2 Département des Sciences Biologiques, Université du Québec à MontréalMontréal, Quebec, Canada

**Keywords:** Behavioral correlations, boldness, personality, predation

## Abstract

Individuals from the same population generally vary in suites of correlated behavioral traits: personality. Yet, the strength of the behavioral correlations sometimes differs among populations and environmental conditions, suggesting that single underlying mechanisms, such as genetic constraints, cannot account for them. We propose, instead, that such suites of correlated traits may arise when a single key behavior has multiple cascading effects on several other behaviors through affecting the range of options available. For instance, an individual's shyness can constrain its habitat choice, which, in turn, could restrict the expression of other behavioral traits. We hypothesize that shy individuals should be especially restrained in their choice of habitat when the risk of predation is high, which then canalizes them into different behavioral options making them appear behaviorally distinct from bolder individuals. We test this idea using an individual-based simulation model. Our results show that individual differences in boldness can be sufficient, under high predation pressure, to generate behavioral correlations between boldness and both the tendency to aggregate and the propensity to use social information. Thus, our findings support the idea that some behavioral syndromes can be, at least to some extent, labile. Our model further predicts that such cascading effects should be more pronounced in populations with a long history of predation, which are expected to exhibit a low average boldness level, compared with predator-naïve populations.

## Introduction

Individuals from the same population generally vary in suites of correlated behavioral traits. For instance, boldness, sociability, aggressiveness, and activity level can be strongly correlated among each other (e.g., Bell and Stamps 2004; Sih et al. 2004; Sih and Bell 2008) as well as with the tendency to rely on social information (Kurvers et al. 2010; Jolles et al. 2013) or dispersal propensity (Dingemanse et al. 2003; Côté and Clobert 2007; Côté et al. 2010). For example, individuals that are the least sociable may happen to be the boldest, most aggressive, least reliant on social information, and most dispersive members of a population. How these trait associations come about has been a subject of increased research, and genetic constraints have arisen as a reasonable underlying mechanism for many of these trait correlations (Brommer and Kluen 2012), thereby assuming that phenotypic correlations between behaviors represent underlying genetic correlations. A genetic correlation can arise, for example, when the same genes affect more than one behavior (i.e., pleiotropy) or when different genes, each coding for different behaviors, are in linkage disequilibrium as the result of selection favoring specific combinations of genes. Recent studies in molecular genetics have provided supporting evidence for the existence of genetic correlations (e.g., Dingemanse et al. 2002; van Oers et al. 2005; Ariyomo et al. 2013).

While genetic correlations can account for many trait correlations, however, they cannot account for all. In particular, those behavioral correlations that exhibit intraspecific variability or that are present in one population but not in another cannot be explained easily by characteristics of a genetic architecture that is shared by all (Bell 2005; Dingemanse et al. 2007; Lacasse and Aubin-Horth 2014; Martins and Bhat 2014). For instance, recent population comparisons in three-spined sticklebacks (*Gasterosteus aculeatus*) show that the association between aggressiveness and boldness differs between populations; the aggressiveness–boldness syndrome exists only in populations that have been subjected to high predation pressure (Bell 2005; Dingemanse et al. 2007). This association between the presence of a behavioral correlation and a specific ecological condition suggests a different, nongenetic type of explanation. Some have pointed to state-dependent effects where the state of the individual (e.g., its size, energy reserves, or condition) determines the benefits associated with a behavior (e.g., Luttbeg and Sih 2010; Wolf and McNamara 2012). Here, we argue instead that such situations may be the result of what we term a “cascading effect” (Dubois et al. 2012). A cascading effect, unlike a state-dependent effect, does not require that ecological conditions alter the adaptive value of other behavioral traits. Instead, cascading effects occur when a given behavioral trait restricts the number of other behavioral options available to individuals. The result is that an ecological condition that calls for the expression of a specific behavioral trait can force individuals into using consistently only a narrower range of behavioral options.

Cascading effects may be common in social organisms where the number of social options available to individuals strongly depends on the presence and location of neighbors. Anything affecting the position of an individual relative to others would then exert cascading effects, for example, on the availability of social information or the need to compete for access to resources. We hypothesize that boldness, defined as the tendency of an individual to take risks (Ward et al. 2004) and/or be exploratory in novel contexts (Wilson and Stevens 2005), can be a good candidate key behavioral trait with the potential to exert cascading effects on many other traits. We know, for instance, that boldness constrains an individual's habitat choice and generates individual differences in sociability (Budaev 1997). We hypothesize that boldness will exert different cascading effects on behavioral traits when animals live under high as opposed to low predation pressure. Explicitly, we assume that low-predation environments would exert no constraints on individuals' habitat choice, whatever their tendency to take risks, and hence should not favor behavioral correlations between boldness and any other trait. Conversely, in high-predation environments, shy individuals, unlike bold individuals, would be strongly limited in the number of suitable habitats they may occupy, thereby making behavioral differences between the two types more pronounced. We test this hypothesis using a simulation model that focuses on the cascading effects that boldness may have on the tendency to aggregate and the availability of social information concerning food. The use of social information is often modelled as a producer-scrounger game (Giraldeau and Dubois 2008). In this game, individuals search for their own food discoveries (producer) or use social information to detect opportunities to join resources uncovered by others (scrounger). So, producer and scrounger serve as indicators of whether individual uses personal or social information, respectively.

## The Model

### General

**Figure 1:**
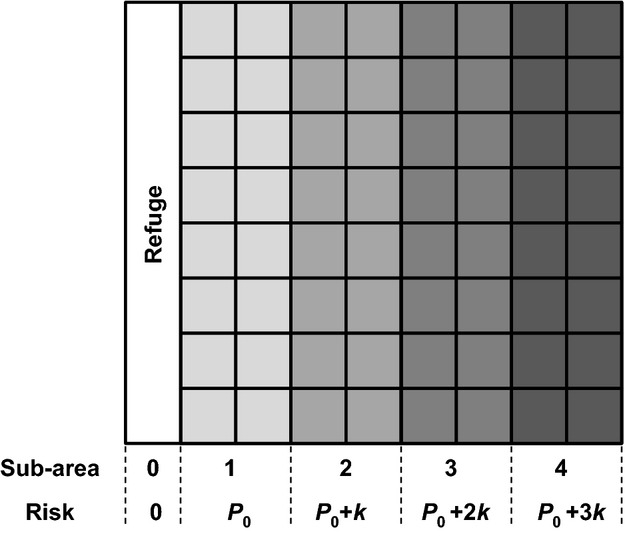
Representation of the foraging area divided into four subareas. The risk associated with each subarea corresponds to the probability of mortality of an isolated individual (i.e., with no neighbor).

The model considers a group of G individuals that search for food on an area divided into (NxN) locations, among which only *n* patches contain F food items. Patches can be exploited at some risk of predation, and a refuge is available to flee predation. We define boldness as the maximum level of risk an individual is willing to take and use two categories of boldness: shy or bold with groups made up of both types of individuals, in proportions *p* and (1−*p*), respectively. An individual's maximum riskiness is fixed and denoted *R*_S_ and *R*_B_ for shy and bold individuals, respectively, with *R*_S _< *R*_B_. The risk of predation depends on both an individual's location in the foraging area and the number of neighbors that dilute his chances of being predated. We consider that the predation risk experienced at a given food patch increases with the patch's distance to the refuge as follows: The whole foraging area is divided into four subareas, and all food patches within a subarea have the same level of riskiness. Patches presenting the lowest risk of predation are those whose *x* coordinate varies between 0 and N/4 and are denoted *P*_0_, increasing in riskiness by *k* from one subarea to the next (Fig. 1).

The number of food patches is kept constant throughout a simulation of *t* steps. Each depleted food patch is then immediately replaced by a new patch containing *F* items and at a position that is randomly selected among all unattended foodless patches.

### Decision-making process

At each time step, individuals, one after the other, decide whether to hide in the refuge or to play either the producer or the scrounger tactic. All individuals use the searching tactic with which they were more successful (Belmaker et al. 2012), and the order at which they make their decision is randomly assigned to ensure that behavioral differences among individuals are unrelated to their decision order. Before making a choice, an animal first assesses the current danger associated with every unattended patch. We consider that the predation risk is reduced when individuals are clumped together and so the danger is calculated as the risk associated with the patch's subarea divided by one plus the number of neighbors present on the adjacent patches. If all patches are beyond the individual's maximum risk threshold (i.e., larger than *R*_S_ or *R*_B_), it goes to the refuge. Otherwise, it either chooses one tactic randomly if the expected success of both tactics is equal, or adopts the tactic with which it was more successful. At the beginning of a simulation, all individuals consider both tactics as equally effective. Then, every time an individual uses a particular tactic, the success of that tactic increases or decreases by one if the animal succeeded or failed in obtaining a food reward, respectively.

When an animal plays producer, it may continue exploiting a previously discovered food patch provided that it is not depleted yet and the current danger on that patch is still acceptable. In all other cases (i.e., the patch is depleted, the location is not sufficiently safe, or the individual had no food reward in the previous step), it goes to a randomly chosen unoccupied patch where the danger is below its acceptance threshold, assesses its quality, and then obtains either one food item or no food.

**Figure 2:**
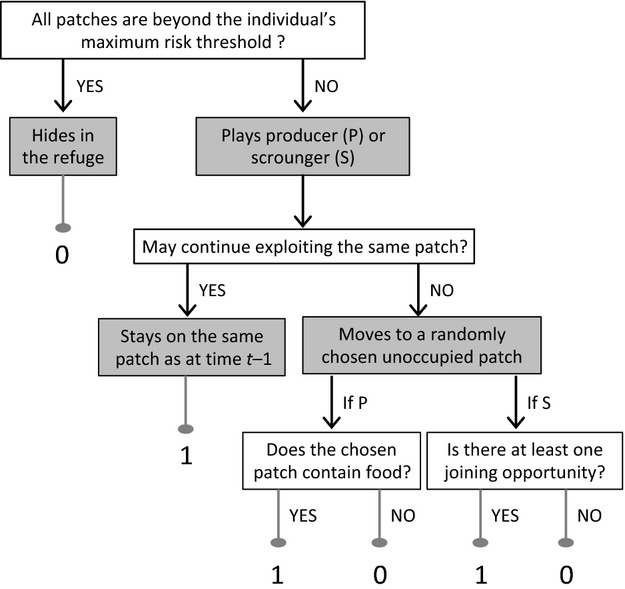
Stages of the decision-making process and gain expected by an individual for each of the six possible cases.

When an animal plays scrounger, it may remain on the same patch and obtain one food item provided that the current danger is acceptable and the patch is not empty. In all other cases (i.e., the patch is depleted, the current location is not sufficiently safe, or the individual had no food reward in the previous step), it moves to a randomly chosen unoccupied patch where the danger is below its acceptance threshold and assesses in a radius of *r* (with 1 ≤ *r *< *N*−1) patches around its current location whether joining opportunities exist. Thus, scrounging opportunities are evaluated for all patches that are within the limits of the foraging area and whose coordinates vary between [*x*−*r*; *x *+ *r*] and [*y*−*r*; *y *+ *r*], where *x* and *y* denote the current position of the focal animal. The animal judges that a patch can be joined if at least one individual, whatever its tactics, is already foraging there and the patch is not depleted. If there is more than one joining opportunity, the animal chooses one randomly and gains one food item. The different stages of the decision-making process are summarized in Fig. 2.

### Analysis

**Figure 3:**
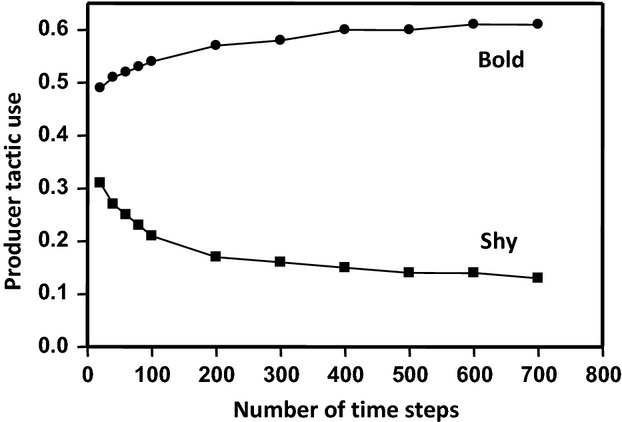
Mean producer tactic use of bold and shy individuals over time. In this figure: *G* = 20, *F* = 10, *n *=* *40, *p *= 0.5, *r *=* *1, *P*_0_ = 0, *k *=* *0.2, *x *=* *0.5, *R*_S__ _= 0.1, and *R*_B__ _= 0.9.

In each simulation, we calculated for every individual the number of steps it spent playing producer and scrounger and its mean number of neighbors, as an index of its tendency to aggregate. Because our model is based on the assumption that clumping reduces predation, we verified that the differences among individuals in their tendency to aggregate were not simply the direct consequence of this assumption by running additional simulations in which the danger associated with every patch was independent of competitor density. For a given set of parameters (Table 1), we ran the simulation 500 consecutive times and computed the average values obtained from each. In addition, because the simulations rapidly reach an equilibrium state (Fig. 3), we ran the simulations during 200 time steps.

**Table 1:** Symbols and their meaning.

Symbol	Meaning
N × N	Dimension of the foraging area (i.e., number of patches); default value = 12 × 12 = 144
*N*	Number of food patches; range of tested values = 10–60
*F*	Patch value; range of tested values = 5–50
*G*	Group size; range of tested values = 10–50
*t*	Simulation length; default value: 200
*R*_B_	Maximum risk that bold individuals are willing to take; range of tested values = 0.1–0.9
*R*_S_	Maximum risk that shy individuals are willing to take; range of tested values = 0.1–0.9
*p*	Proportion of bold individuals; range of tested values = 0.1–0.9
*P*_0_	Risk of predation associated with patches that are located in the closest subarea to the refuge; range of tested values = 0–0.2
*k*	Increase in predation risk from one subarea to the next; range of tested values = 0–0.3
*r*	Maximal distance at which individuals can perceive scrounging opportunities; range of tested values = 1–11

## Results

**Figure 4:**
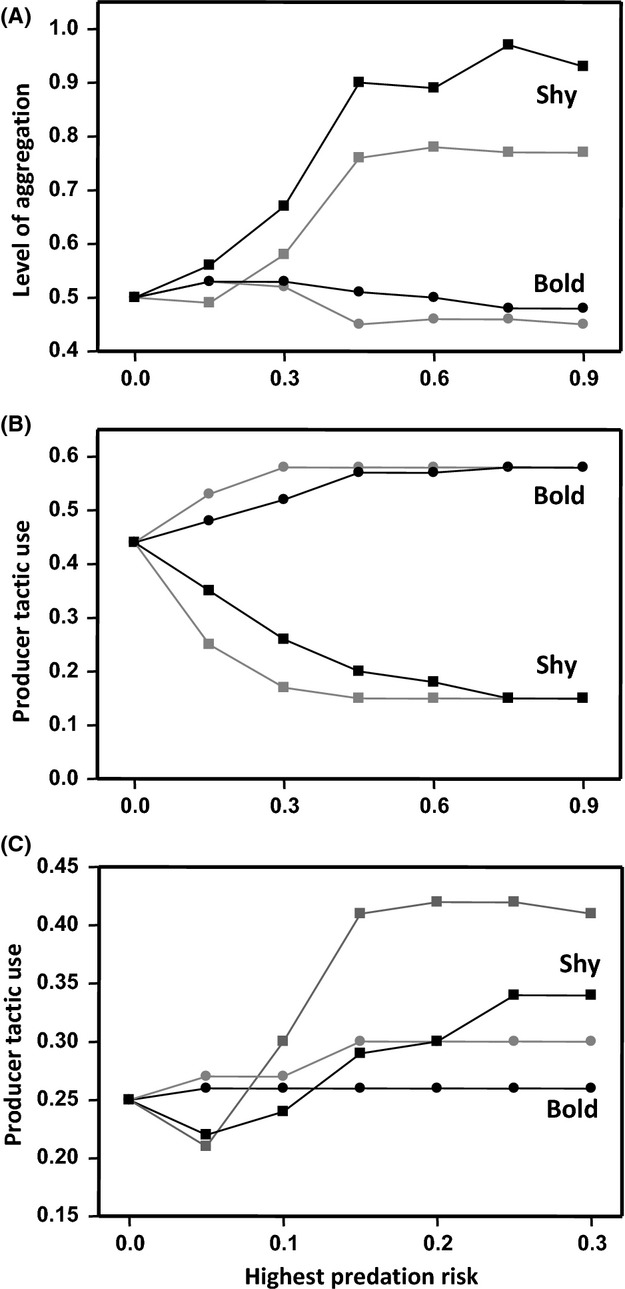
Mean level of aggregation based on the mean number of neighbors (panel A) and producer tactic use (panels B and C) of bold (circles) and shy (squares) individuals in relation to predation pressure. The maximum distance at which individuals can detect scrounging opportunities is *r *=* *1 (panel B) or *r *=* *11 (panel C). The dark and gray symbols correspond to the case where the danger is dependent or independent on competitor density, respectively. In this figure: *G* = 20, *F* = 10, *n *=* *40, *p *=* *0.5, *P*_0_ = 0, *x *=* *0.5, *R*_S__ _= 0.1, and *R*_B__ _= 0.9.

When the predation danger is low in all subareas, even those far from a refuge, the model predicts no correlation between boldness and aggregation tendency. Conversely, when the predation risk rapidly increases with distance to cover, shy individuals prefer to forage in subareas closer to cover, where the resulting density of individuals becomes higher than in other subareas. Under high predation risk, therefore, because they prefer the same set of subareas, shy individuals will appear more sociable than bold individuals (Fig. 4A) and so a behavioral correlation emerges. The strength of the correlation between boldness and the use of the producer tactic also increases with the risk of predation (Fig. 4B,C), but the relationship between the preferred tactic and boldness level depends on the distance at which scrounging opportunities can be detected. When only short-distance opportunities are detectable, shy – more highly aggregated – individuals will have a greater chance of detecting scrounging opportunities than bold individuals that are generally further apart, and so shy individuals will scrounge more than the bold individuals (Fig. 4B). In contrast, when all scrounging opportunities are detectable, irrespective of distance, shy individuals will produce more than bold individuals (Fig. 4C) because bold individuals are now as effective as shy individuals at detecting scrounging opportunities. However, as predation increases, shy individuals become increasingly constrained in their choice of foraging sites, and as a result, they are exposed to fewer scrounging opportunities than bold individuals who remain capable of exploiting all sites despite the increased predation. The same pattern emerges when a patch's level of danger is independent of competitor density (Fig. 4). These findings show that the tendency for shy individuals to clump under high predation pressure may not be due to dilution of predation pressure in groups but rather to their avoidance of risky foraging sites, which, in turn, forces them to forage in higher density in the few safer areas closer to cover.

**Figure 5:**
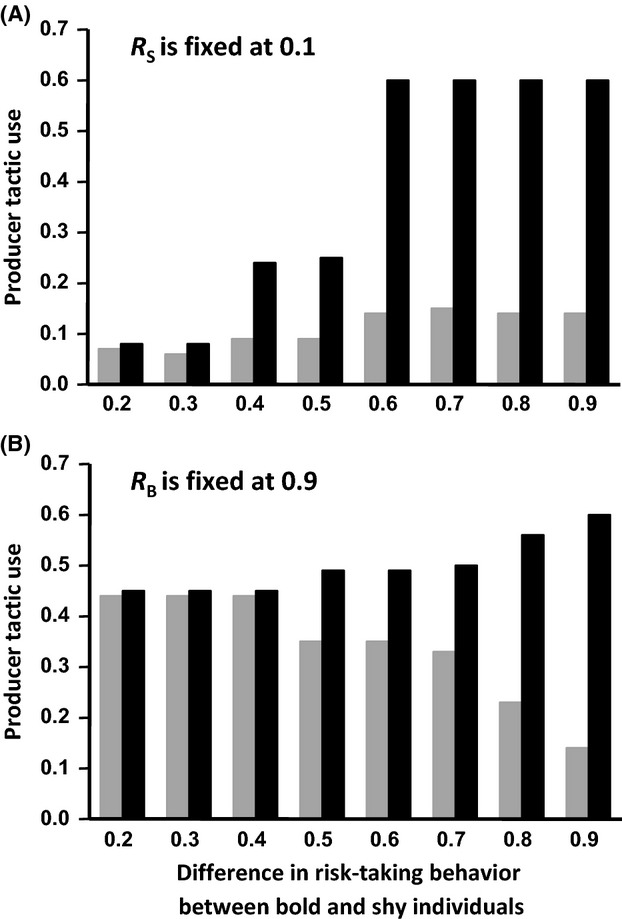
Mean producer tactic use of bold (dark bars) and shy (gray bars) individuals, in relation to the difference in the risk-taking behavior between the two behavioral types when the average level of boldness is low (panel A) or high (panel B). In this figure: *G* = 20, *F* = 10, *n *=* *40, *p *=* *0.5, *P*_0_ = 0, *k *=* *0.2, *r *=* *1, and *x *=* *0.5.

**Figure 6:**
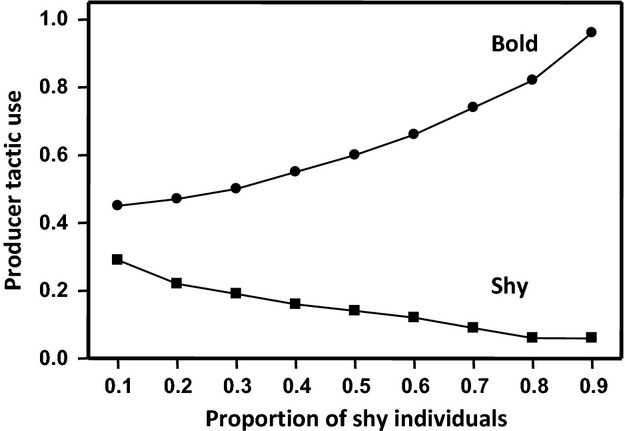
Mean producer tactic use of bold and shy individuals in relation to the proportion of shy individuals. In this figure: *G* = 20, *F* = 10, *n *=* *40, *p *=* *0.5, *r *=* *1 and *P*_0_ = 0, *R*_S__ _= 0.1 and *R*_B__ _= 0.9.

Under high predation pressure, the differences between bold and shy individuals in their propensity to use the producer tactic are more pronounced when food patches are poor (i.e., small values of F) and the number of competitors is small. By contrast, the correlation between boldness and foraging tactic use is not affected by patch richness or group size under conditions of low predation risk. Similarly, under high predation pressure, boldness has a stronger effect on the tendency to aggregate in small groups compared with large ones and when the number of food patches is small. However, none of these factors significantly affect the strength of the correlation between boldness and the tendency to aggregate when the danger is low in all subareas. Finally, the effects of boldness on aggregation level and foraging tactic use are influenced by the average level and variance of boldness. The differences between bold and shy individuals in terms of their tendency to aggregate and propensity to use the producer tactic increase with the difference between their maximum risk-taking thresholds, but decrease as the average level of boldness increases (Fig. 5). The cascading effects of boldness are then stronger (1) when the two behavioral types differ widely from each other in their acceptance threshold and (2) for a fixed threshold difference, when all individuals are willing to take relatively few risks compared to when both behavioral types explore their environment more thoroughly. For the same reason, the cascading effects of boldness on the tendency to aggregate and producer tactic use are expected to be more pronounced within groups composed mainly of shy individuals (Fig. 6).

## Discussion

Our simulation model supports our hypothesis that labile behavioral correlations can arise from nongenetic means when some key behavior, under some specific ecological circumstances, exerts cascading effects on the range of behaviors available to individuals. Our simulation model shows that boldness can be one such key behavior exerting its cascading effects on both aggregation tendency and foraging tactic use under conditions of high predation pressure.

Our study is not the first to point out the possibility of behavioral correlations without an underlying genetic constraint. For instance, Wolf and McNamara (2012) describe a behavioral correlation when the correlated traits are affected by the same underlying physiological mechanism: metabolic rates. These rates are correlated with differences in aggressiveness and foraging tactic use, because the physiological state of the individuals affects the costs and benefits of behavioral alternatives in both contexts, and individuals therefore are expected to act consistently across contexts and situations. Our model, however, goes much further than Wolf and McNamara's situation because in our case, individual differences in boldness have no direct effect on the payoffs associated with either behavioral tactics or on the individuals' decision-making process. Instead, the behavioral correlations emerge because the tendency of individuals to take risks affects their spatial distribution, which, in turn, determines the scope of behavioral options available to them. Thus, our findings demonstrate that behavioral correlations may arise entirely through behavior without the need for underlying genetic, physiological, or cognitive constraints.

Compared with positive feedback mechanisms that provide a powerful explanation for behavioral correlations that are maintained over the long term (Sih and Bell 2008; Wolf et al. 2008; Luttbeg and Sih 2010; Wolf and Weissing 2010), the cascading effects illustrated by our simulation can account for a number of reported situations where behavioral correlations are either condition dependent (Bell 2005; Bell and Sih 2007; Dingemanse et al. 2007) or trait dependent (Garamszegi et al. 2012). More precisely, state-dependent models have demonstrated that differences among individuals both in their state and in their behavioral traits can be maintained over the long term only if there are positive feedbacks between the benefits associated with a particular behavior and the state of the animal. Otherwise, both behavior and state converge, leading to a decrease in the strength of the correlations (see Dingemanse and Wolf 2010). Feedback mechanisms therefore cannot account for behavioral correlations that are sensitive to environmental conditions. Conversely, consistent with our expectation, we found that the strength of the behavioral correlations between boldness and both aggregation tendency and foraging tactic use was affected by predation risk: Under low predation risk, dispersal tendency did not differ between bold and shy individuals that, consequently, did not differ in their level of aggregation or propensity to use social information either. In contrast, under higher predation pressure, shy individuals had a higher tendency to stay close to cover, thereby generating differences in aggregation level and foraging tactic use among individuals that were associated with differences in their risk-taking behavior. The cascading effects can therefore explain not only why populations may differ in the strength of behavioral correlations, but also why exposure to predation in particular can generate such correlations (Bell and Sih 2007).

Our results further suggest that predation pressure would have an additional effect on the existence of a behavioral syndrome by affecting the mean level of boldness in the population. Indeed, our simulation model revealed that, under a similar risk of predation, the strength of the correlations decreased when the average level of boldness increased. Whatever the mechanism (e.g., frequency dependence) that maintains genetic variation among individuals in their level of boldness, selection should favor being bolder in low-risk situations (Brown et al. 2005; Chiba et al. 2007). Hence, we would expect the type of behavioral correlations we studied here to occur more frequently in populations with a long history of predation compared with predator-naïve populations, regardless of the current predation pressure in line with observations by Lacasse and Aubin-Horth (2014). In that recent study, the authors report that individual aggressiveness was negatively correlated with sociability in juvenile sticklebacks, but only for the population from a high predation risk. Conversely, in juveniles that came from a population with no predators, the traits were not correlated, although sticklebacks from both populations had been reared in a common predator-free environment (Lacasse and Aubin-Horth 2014).

An increase in predation pressure increased the strength of the correlation between boldness and foraging tactic use, but the sign of the correlation was determined by the distance at which individuals can detect scrounging opportunities. When individuals can perceive all scrounging opportunities, whatever the distance, bold individuals rely more on social information compared with shy individuals under high predation risk. Alternatively, when only short-distance opportunities are detectable, the difference between bold and shy individuals in scrounger tactic use decreases with increasing predation pressure. Because the scrounging horizon (i.e., the maximum distance at which individuals can detect feeding companions) likely depends on some specific (e.g., cognitive abilities) and ecological (e.g., vegetation density) characteristics, our results indicate that the cascading effects of boldness on social information use should vary among both species and conditions. Furthermore, under conditions of high predation risk, the strength of the correlation between boldness and foraging tactic use (but not aggregation level) is affected by the richness of food patches. This finding suggests that even if a single trait (i.e., boldness) has multiple cascading effects on several other behaviors, they do not necessarily all respond in the same way to environmental changes.

Our findings support the empirical evidence that individual differences in boldness are generally associated with individual differences in plasticity, with shy individuals being more flexible than bold individuals (Benus et al. 1991; Koolhaas et al. 1999). Accordingly, we found that an increase in predation risk had no or little effect on the behavior of bold individuals, but resulted in marked changes in both the level of aggregation and the use of the producer tactic of shy individuals. Behavioral plasticity is often considered beneficial (Wilson 1998) but costly (DeWitt et al. 1998), which may explain why fixed individuals are maintained within populations. Another explanation for this polymorphism is that individual differences in a single key trait like boldness through cascading effects limit the magnitude of the response to environmental changes. For instance, given that bold individuals take more risks than shy individuals, they tend to disperse more randomly relative to danger even when predation pressure is high and, consequently, exhibit lower levels of aggregation in conditions expected to favor aggregation. This mechanism results in bold individuals being less responsive to changes in predation pressure compared with shy individuals.

In conclusion, extensive work on animal personality over the last decade has identified that animals within a population generally differ not only in (1) single behaviors but also in (2) suites of correlated behavioral traits as well as in (3) their level of phenotypic plasticity (Dingemanse and Wolf 2010). Most theoretical studies have concentrated only on a single pattern of those differences. However, our study demonstrates that fixed individual differences in a single trait may favor tight associations among several behaviors as well as consistent individual differences in plasticity, thereby suggesting that the different patterns of variation should not be treated as separate issues. Furthermore, taken together, our findings suggest that behavioral syndromes would be, at least to some extent, labile (Bell and Stamps 2004; Bell and Sih 2007; Dingemanse et al. 2007; Cote et al. 2013) and hence would not impose evolutionary constraints on the course and outcome of evolution (Wolf and Weissing 2012).
